# Motor engagement enhances incidental memory for task-irrelevant items

**DOI:** 10.3389/fpsyg.2022.914877

**Published:** 2022-08-16

**Authors:** Daisuke Shimane, Takumi Tanaka, Katsumi Watanabe, Kanji Tanaka

**Affiliations:** ^1^Research Center for Brain Communication, Kochi University of Technology, Kochi, Japan; ^2^Graduate School of Humanities and Sociology and Faculty of Letters, The University of Tokyo, Tokyo, Japan; ^3^Faculty of Science and Engineering, Waseda University, Tokyo, Japan; ^4^Faculty of Arts and Science, Kyushu University, Fukuoka, Japan

**Keywords:** episodic memory, action execution, action preparation, action-induced memory enhancement, attentional boost effect

## Abstract

Actions shape what we see and memorize. A previous study suggested the interaction between motor and memory systems by showing that memory encoding for task-irrelevant items was enhanced when presented with motor-response cues. However, in the studies on the attentional boost effect, it has been revealed that detection of the target stimulus can lead to memory enhancement without requiring overt action. Thus, the direct link between the action and memory remains unclear. To exclude the effect of the target detection process as a potential confounder, this study assessed the benefit of action for memory by separating items from the response cue in time. In our pre-registered online experiment (*N* = 142), participants responded to visual Go cues by pressing a key (i.e., motor task) or counting (i.e., motor-neutral cognitive task) while ignoring No-go cues. In each trial, two task-irrelevant images were sequentially presented after the cue disappearance. After encoding the Go/No-go tasks, participants performed a surprise recognition memory test for those images. Importantly, we quantified the impact of overt execution of the action by comparing memories with and without motor response and the impact of covert motor processes (e.g., preparation and planning of action) by comparing memory between the motor and cognitive tasks. The results showed no memory differences between Go and No-go trials in the motor task. This means that the execution itself was not critical for memory enhancement. However, the memory performance in the motor No-go trials was higher than that in the cognitive No-go trials, only for the items presented away from the cues in time. Therefore, engaging the motor task itself could increase incidental memory for the task-irrelevant items compared to a passive viewing situation. We added empirical evidence on the online interaction between action and memory encoding. These memory advantages could be especially brought in action preparation and planning. We believe this fact may expand our present understanding of everyday memory, such as active learning.

## Introduction

Episodic memory is a core function of cognition and has been one of the most classic and popular topics in empirical psychology and neuroscience. In these fields, researchers have typically measured one's memory in passive situations (Heuer et al., 2020), wherein participants were asked to see stimuli without intentional body movements to minimize noises in the data. In reality, however, humans constantly explore the external world *via* their movements. The actions strongly influence what we experience and what information is encoded. Literature has demonstrated how concurrent actions shape our perception (Nicolelis et al., 1996; Zwickel et al., 2007; Liu et al., 2008; Kirsch, 2015; Kumar et al., 2015; van Ede et al., 2015; Arikan et al., 2017; Heuer and Schubö, 2017; Schneider et al., 2017; Yon et al., 2018; Gallivan et al., 2019), attention (Hannus et al., 2005; Baldauf et al., 2006; Baldauf and Deubel, 2008), and working memory (Boon et al., 2014; Hanning and Deubel, 2018; van Ede et al., 2019). Moreover, in the framework of active learning, educators have augured the benefit of active involvement in memory (Michael, 2006). Nevertheless, there has been insufficient empirical evidence on the online interaction between action and memory.

A recent work by Yebra et al. (2019) shed light on this issue and reported the memory advantage of actions. In their experiments, participants first performed a motor Go/No-go task for incidental encoding. In the task, a rectangle frame and a grayscale image of a daily object in the frame were simultaneously presented at random intervals. The frame was colored in one of the two colors. Depending on the frame color, participants either executed or withheld a keypress (Go and No-go trials, respectively). The images were task-irrelevant; participants were instructed to just look at them. In the surprise recognition memory test, participants remembered images presented in the Go trials better than images presented in the No-go trials. Yebra et al. (2019) referred to this advantage in memory performance by active involvement as the “action-induced memory enhancement” (AIME).

The relationship between action and memory processes was further explored by Kinder and Buss (2020). In their experiment, participants pressed a key when the images of a face of specific sex were presented while ignoring the face of the other sex. Kinder and Buss (2020) employed a cognitive Go/No-go task as a motor-neutral baseline, in which participants counted every presentation of the target sex. Here, the face images were response cues, as well as study items and, thus, were task-relevant. The memory performance for the face images was compared among the motor Go, motor No-go, and motor-neutral conditions. The authors considered the difference between motor Go and motor No-go items to represent the impact of action execution, whereas that between motor No-go and motor-neutral baseline to represent the covert motor processes that include action preparation and inhibition. These contrasts suggested that both action execution and covert motor processes could contribute to memory enhancement. Kinder and Buss (2020) collectively referred to these overt and covert processes as motor engagement.

In contrast, another body of work provides an alternative explanation for the interaction of action and memory. Attention research reported a similar memory enhancement, that is, a memory advantage for items presented concurrently with to-be-responded targets compared to those presented with distractors. Named the “attentional boost effect” (ABE; Swallow and Jiang, 2010), this effect is thought to occur because attention to targets *spills over into* peripheral stimuli and facilitates encoding (Swallow and Jiang, 2013). This means that target detection, not motor engagement, is critical for ABE. Indeed, ABE can be elicited not only by the motor response but also the cognitive response, like counting the targets (Swallow and Jiang, 2012; Makovski et al., 2013; Toh and Lee, 2022). This fact casts a doubt on the direct link between action execution and memory encoding. Given that the items were presented simultaneously with the behavioral targets, better memory for the Go items in Yebra et al. (2019) may be attributed to attention to the targets, not the motor response^1^. A similar interpretation is also plausible for Kinder and Buss (2020), where the motor Go items were response cues themselves. On the contrary, better memory for motor No-go vs. motor-neutral items cannot be explained by ABE. Although this could support the motor-specific enhancement, such a role of the covert process has not yet been explored with incidental memory for the task-irrelevant items.

As such, the pure impact of concurrent motor engagement on task-irrelevant memory is still unclear. To solve this problem, this study attempted to separate the AIME from ABE. Swallow and Jiang (2011) manipulated the temporal asymmetry between a response cue and a study item and observed the ABE only for the item presented simultaneously with the cue, leaving the item presented before or after it. This allowed us to reason that, if enhancement occurred for items presented after the disappearance of cues, the memory enhancement should not be attributed to the known ABE. Therefore, we investigated whether motor engagement would cause memory enhancement, even when the items were temporally separated from the behavioral targets (i.e., Go cue). Naturally, such separation from target detection is possible only when study items are task-irrelevant. Excluding the potential confounding with ABE enabled us to assess the influence of motor engagement on memory. Specifically, we explored the impact of the overt process on memory by comparing Go and No-go in the motor task, and the impact of the covert process on memory by comparing the motor task with the cognitive task as the motor-neutral baseline.

Accordingly, we conducted a pre-registered online experiment (*N* = 142) and employed motor and cognitive Go/No-go tasks for encoding. In these tasks, we sequentially presented the Go/No-go cues and grayscale images of daily objects without temporal overlap. Moreover, following the presentation of the Go/No-go cues, we presented the images at two different periods, separated by the action execution. Yebra et al. (2019), as well as our previous work (Shimane et al., 2021), indicated the presence of a temporal window of memory enhancement; the effect of the action was relatively unreliable for items presented before action execution. Considering such a confounding influence, we separately evaluated the memories before and after the action execution. In the motor Go trial, the pre-action image was presented after the Go cue disappeared. Then, the participants' keypress caused the disappearance of the pre-action image and the presentation of the post-action image. In the motor No-go trial, the pre-action image automatically replaced the post-action image without a keypress. The sequence of events was the same for the cognitive task except the participants counted the number of Go cues instead of pressing a key. Finally, we compared the incidental memory for those images between conditions.

## Materials and methods

### Participants

We used a web-based power application PANGEA (v0.2) (https://jakewestfall.shinyapps.io/pangea/) to conduct an *a priori* power analysis. Assuming the effect size of the action on memory observed in our previous work (*d* = 0.27; Shimane et al., 2021), we calculated the sample size to achieve 0.95 power at the standard 0.05 alpha error probability. This analysis revealed that 129 participants and 12 observations per condition were needed. As this experiment would be performed online, we predicted that several participants' data may need to be excluded. We thus recruited 162 participants to achieve 0.99, 0.95, or 0.80 power when 100%, 80%, or at least 50% of samples would be available, respectively. As mentioned below, we finally analyzed the data from 142 participants (80 women, mean age ± standard deviation (SD) = 40.27 ± 9.55). The participants were recruited *via* an online crowdsourcing service (CrowdWorks; https://crowdworks.jp/).

### Material

We used grayscale images of icons that depict everyday objects (e.g., books, cars, clocks, etc.). The 288 images were collected from an open online database (https://icooon-mono.com). The images were divided into two pre-determined datasets, which were assigned to study (old) and non-study (new) items in a counterbalanced way across participants (i.e., 144 items each).

### Procedure

#### Encoding phase

**Figure 1:**
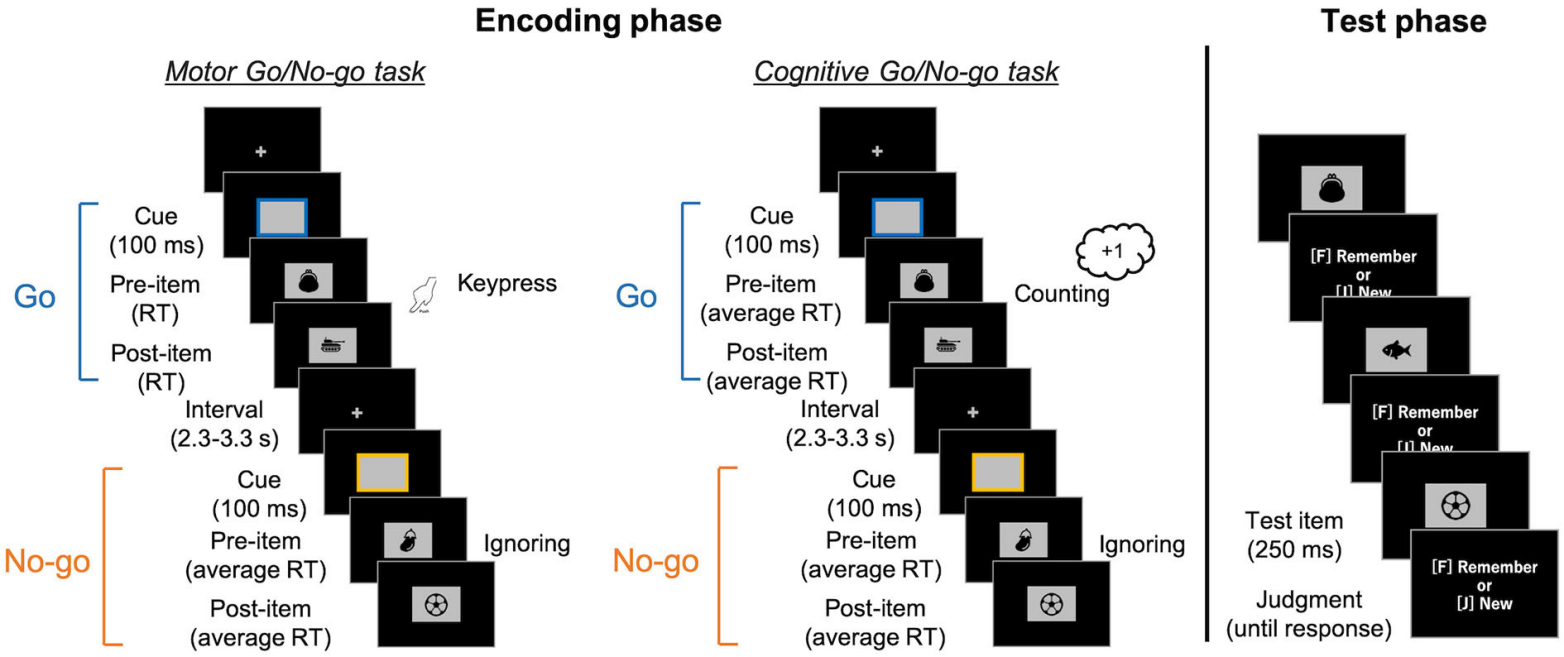
Schematic illustrations of the encoding Go/No-go tasks (left) and the surprise recognition memory test in both (right). In the encoding tasks, the colored frames (i.e., response cue) were presented for 100 ms, with random intervals varying between 2.3 and 3.3 s. Participants were asked to press a key (in the motor task) or count it (in the cognitive task) when the Go cue with a particular color was presented. After the cue disappeared, a pre-item was presented. In the Go trials of the motor task, the pre-item was presented until participants' keypress responses. It was then replaced by a post-item. As the post-item was presented for the response time (RT) in that trial, the presentation duration was the same for pre- and post-items. In the other conditions, pre-items initially appeared for a pre-recorded average RT. They were then replaced by post-items for the same amount of time. Next, they engaged in a simple calculation task for 1 min. Finally, in the recognition memory test, images comprising old and newly added items were presented and participants judged whether they were presented in the encoding task.

Two types of tasks were provided in the encoding phase: motor and cognitive Go/No-go tasks (Figure 1). These tasks were conducted in separate blocks of eight trials, and participants were instructed on which task would be carried out at the beginning of each block.

In both tasks, rectangular frames as response cues were presented for 100 ms at the center of a black screen, with random intervals between 2.3 and 3.3 seconds. The cue was randomly colored either blue or yellow at equal frequencies. In the motor task, participants responded to the Go cues with a particular color (e.g., the blue cues) with a keypress while ignoring the No-go cues (e.g., the yellow ones). In the cognitive task, they counted the Go cues instead of keypress and reported the total number at the end of the block. Every frame was followed by the presentation of two task-irrelevant images. The first image (i.e., pre-item) appeared simultaneously with the cue disappearance. In motor Go trials of the motor task (i.e., motor Go trials), the participants' keypress terminated the presentation of the pre-item and initiated the presentation of the second image (i.e., post-item) at the same location. The post-item was presented for the same duration as the pre-item; that is, the response time (RT) of keypress in that trial. In the other three conditions (i.e., motor No-go, cognitive Go, and cognitive No-go trials), a pre-item appeared for average RT of keypresses in past motor Go trials, and then the post-item was presented for the same amount of time. The average RT was updated in every motor Go trial. The study items were randomly assigned into pre/post condition and motor/cognitive tasks. The assignment of images to the colors of the frame was counterbalanced across the participants.

Participants first practiced the motor and cognitive Go/No-go tasks for 16 (eight each) trials, each without presentation of the images. Then, participants performed six blocks of the motor Go/No-go task and three blocks of the cognitive one with item presented in a random order^2^. Each block comprised one of the combinations of five Go and three No-go, four Go and four No-go, or three Go and five No-go trials. Participants were asked to simply keep looking at the center of the screen where a fixation cross appeared, as well as to not be concerned if the image sometimes appeared there.

#### Test phase

After the encoding phase, participants engaged in a simple calculation task composed of multiplications of two-digit numbers for 1 min. Then, participants performed a surprise recognition memory test. The 288 images, containing 144 old and 144 new items, were serially presented. Participants judged whether each image has been presented in the Go/No-go task with keypresses without time limitation (F-key for “old” and J-key for “new” responses). The images were presented for 250 ms in a random order, to ensure that their duration was the same in the encoding and test phases as in Yebra et al. (2019).

The experiment was implemented with PsychoPy (Peirce, 2007) and provided *via* the Pavlovia platform (https://pavlovia.org). The experiment was conducted on a web browser with a JavaScript application jsPsych (de Leeuw, 2015).

### Data analysis

In each condition, we calculated the rates of “old” responses for old items (i.e., hit rate) and that for new items (i.e., false alarm rate). As in Yebra et al. (2019), we used the difference between a hit and false alarm rates as a proxy for memory performance. Higher hit rates, compared to false alarms, indicate better memory. We also calculated the d-prime and criterion for each condition with the signal detection analysis and reported them in the Supplementary Tables S1.

We excluded the data from participants who 1) provided inaccurate responses in more than 10% of all trials of the motor task, 2) provided two or more inaccurate counting responses in the cognitive task, and 3) displayed memory performance below chance level (i.e., less than zero) in the memory test.

As they were pre-registered, we assessed the memory difference between Go and No-go trials in all conditions composed of task types and item presentation onsets by two-tailed *t*-tests and effect size estimation. We quantified the effect size (*d*) with non-parametric bootstrapped estimation (Halsey et al., 2015; Halsey, 2019). This estimation analysis could supplement significance tests in effect sizes and relative precision (Claridge-Chang and Assam, 2016; Ho et al., 2019b).

We planned that when there was no memory difference between Go and No-go trials in the cognitive task, we would perform a two-way within-participants analysis of variance (ANOVA) by lumping them together as the motor-neutral condition (cf., Kinder and Buss, 2020). The independent variables were item presentation onset (pre or post) and motor engagement (motor Go, motor No-go, or motor-neutral), whereas the dependent variable was memory performance. However, combining two heterogeneous conditions into one would lead to the wrong conclusion. Thus, when there was a significant memory difference between Go and No-go trials in the cognitive task, we orthogonally assessed the effect of cue type and task type instead of aggregating them. In such a case, we conducted a three-way within-participants ANOVA, including the task type, as an additional independent variable. The independent variables were item presentation onset (pre or post), cue type (Go, No-go), and task type (motor or cognitive). The statistical analysis and data visualization were conducted using R [Version 4.1.2; R Core Team (2021)] and the R-packages *dabestr* [Version 0.3.0; Ho et al. (2019a)], *ggplot2* [Version 3.3.5; Wickham (2016)], and *reticulate* [Version 1.24; Ushey et al. (2022)]. The *p* < 0.05 (two-tailed) were deemed significant.

### Ethical considerations

This study was approved by the institutional review board of the University of Tokyo (no. 202119) and conducted in accordance with the ethical standards of the 1964 Declaration of Helsinki. All participants provided informed consent before the commencement of experiments.

## Results

Five participants were excluded from the analysis because of low performance in the motor (*n* = 2), cognitive (*n* = 2), or both Go/No-go tasks (*n* = 1). After rejecting these participants' data, the mean ratio ± SD of correct motor Go/No-go responses was 99.80 ± 0.73%, and the mean RT ± SD in the motor Go trials was 293.35 ± 69.95 ms. Moreover, data from 15 participants were excluded due to extremely low memory performance in the test. Thus, the data from 142 participants were analyzed.

As planned, we first assessed the difference in memory performance between Go and No-go conditions. The *t*-tests detected a significant difference only for the post-items in the cognitive task [*t*_(141)_ = 2.08, *p* = 0.039, *d* = 0.19, 95% confidence interval (CI) = (−0.04, 0.42)]. In the cognitive task, the post-items presented in the Go trials were better remembered than No-go trials. There was no significant difference in pre-items in the cognitive task [*t*_(141)_ = 0.32, *p* = 0.746, *d* = 0.03, 95% CI = (−0.19, 0.28)] and pre- [*t*_(141)_ = 1.15, *p* = 0.254, *d* = 0.10, 95% CI = (−0.13, 0.34)] and post-items [*t*_(141)_ = 1.20, *p* = 0.232, *d* = −0.10, 95% CI = (−0.33, 0.14)] in the motor task.

**Figure 2:**
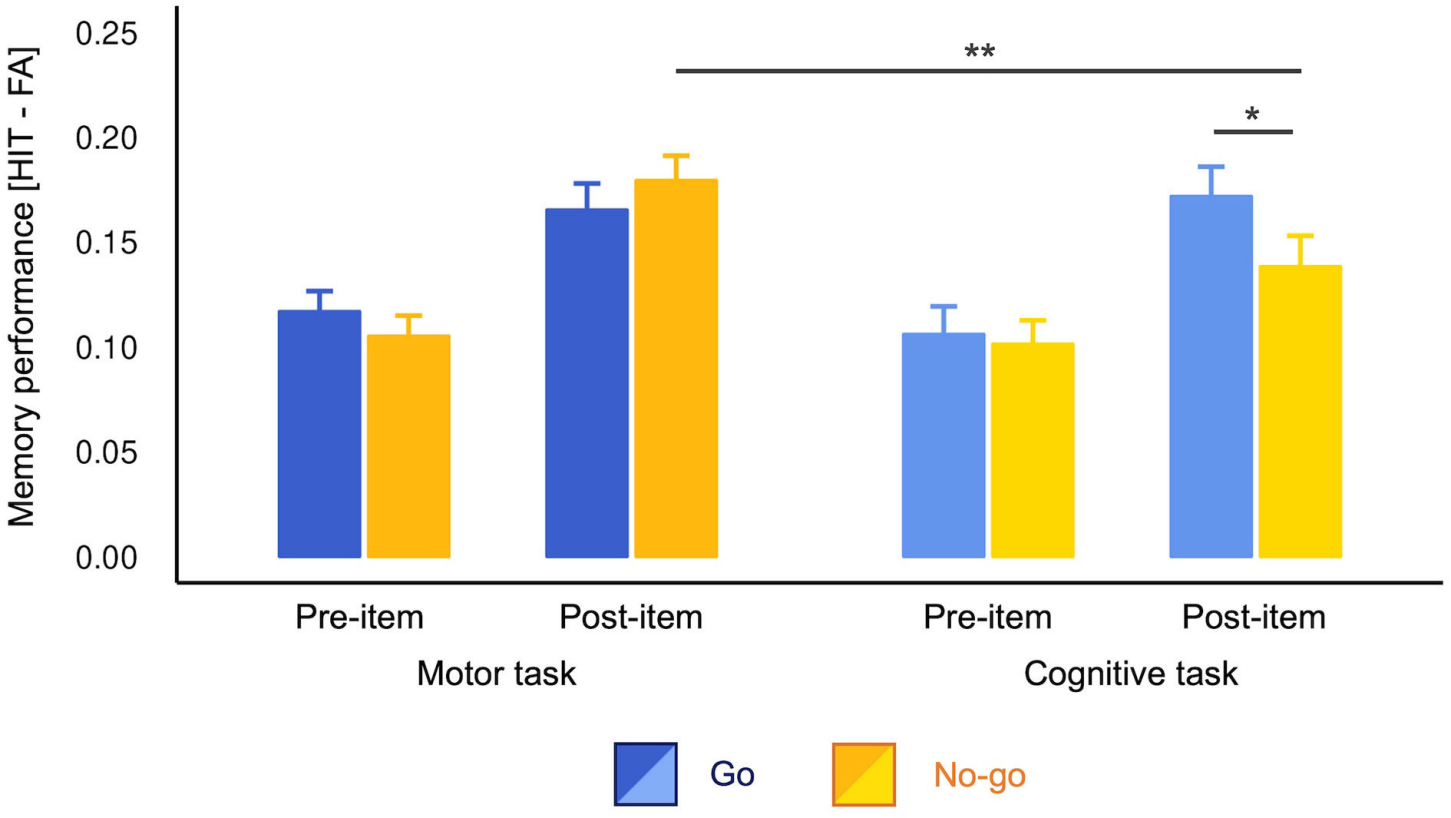
Memory performance across conditions. Performance was calculated by subtracting the false alarm rates from the hit rates. The No-go post-items were better memorized in the motor task than in the cognitive task. The Go post-items were better memorized than No-go post-items in the cognitive task only in the motor cognitive task. The error bars depict standard errors of measurement. *p < 0.05, **p < 0.01.

As we observed the difference between Go and No-go conditions in the cognitive task, we conducted a three-way ANOVA without lumping them together. The results revealed a significant three-way interaction [*F*_(1, 141)_ = 4.05, *p* = 0.046, ${\hat{\eta }}_{G}^{2}=0.002$]. Consistent with the *t*-tests above, the *post hoc* analyses revealed simple-simple main effects of cue type only for post-items in the cognitive task [*F*_(1, 141)_ = 4.35, *p* = 0.039, ${\hat{\eta }}_{G}^{2}=0.009$; Figure 2]. Again, there was no significant Go vs. No-go difference in other three conditions [cognitive pre-item: *F*_(1, 141)_ = 0.11, *p* = 0.746, ${\hat{\eta }}_{G}^{2}=0.000$, motor pre-item: *F*_(1, 141)_ = 1.31, *MSE* = 0.01, *p* = 0.254, ${\hat{\eta }}_{G}^{2}=0.003$, motor post-item: *F*_(1, 141)_ = 1.44, *p* = 0.232, ${\hat{\eta }}_{G}^{2}=0.002$]. Furthermore, in the Go trials, simple-simple main effects of task type were non-significant for both pre- [*F*_(1, 141)_ = 0.68, *p* = 0.409, ${\hat{\eta }}_{G}^{2}=0.002$] and post-items [*F*_(1, 141)_ = 0.21, *p* = 0.644, ${\hat{\eta }}_{G}^{2}=0.000$]. These results indicate that action execution itself modulated neither pre- nor post-item memory. However, we found a simple-simple main effect of task type for No-go post-items [*F*_(1, 141)_ = 9.39, *p* = 0.003, ${\hat{\eta }}_{G}^{2}=0.017$]. That is, No-go post-items were better memorized in the motor task than in the cognitive task, indicating that motor engagement promoted memory encoding compared to the motor-neutral baseline. Simple-simple main effects of onset for all conditions were also significant (post-items > pre-items; *Fs* > 7.169, *ps* < 0.008). There were no other significant simple-simple main effects (*ps* > 0.05). For more detailed results concerning this ANOVA, see Supplementary Table S2.

## Discussion

We investigated whether motor engagement would enhance incidental memory for task-irrelevant items. Specifically, we examined whether overt action execution and/or covert motor processes would enhance memory for the task-irrelevant items presented separately from the behavioral cues. Consequently, we observed no memory differences between Go and No-go trials in the motor task nor between the motor and cognitive Go trials. The AIME reported in Yebra et al. (2019) was not replicated, at least, in its original form. Our results suggested that action execution itself was not critical for memory enhancement. Given that the items were presented simultaneously with behavioral cues in Yebra et al. (2019), the influence of action execution might be confounded with that of target detection. However, only for the items presented away from the cues in time, did we find higher memory performance in the motor No-go trials than that in the cognitive No-go trials. This suggested that motor engagement, especially the covert motor processes, such as action preparation and planning, enhanced the memory encoding. These results are consistent with previous studies on task-relevant memory (Chiu and Egner, 2015a,b; Kinder and Buss, 2020). While denying the advantage of action execution, this study first specified the memory difference between the motor and cognitive No-go trials, thereby clarifying the contribution of the covert motor process to task-irrelevant memory.

Following Swallow and Jiang (2011), we assumed that ABE would not occur by temporally separating the study items from the response cues. However, in the cognitive task, the memory for the post-item was higher in the Go than in the No-go trials. This effect might be attributed to the target detection process as in ABE that there is a possibility that the target presentation enhances memory even after its disappearance in some situations. For example, if a cue and an item are presented with a short time lag, attention may be transiently suppressed after target detection (e.g., attentional blink; Raymond et al., 1992), impeding memory encoding^3^. Indeed, the memory for pre-items was generally inferior to that for post-items, in our experiment, implying the possibility that target detection interfered with the encoding of the pre-items but not the post-items. If this account is the case, target presentation may facilitate memory for an item presented after the suppression period. Otherwise, the ABE-like effect for the post-item might be specific to incidental memory. While we employed the surprise memory task, prior studies on ABE, including Swallow and Jiang (2011), typically used an intentional memory task. Under incidental encoding situations, previous studies yielded mixed evidence about the occurrence of ABE (Swallow and Jiang, 2011; Spataro et al., 2013; Turker and Swallow, 2019; Broitman and Swallow, 2020; Hutmacher and Kuhbandner, 2020). Although it is debatable whether the enhancement that we observed in the cognitive task has the same basis as the known ABE, our results may cast doubt on the limited temporal window and requirement of intentionality in ABE.

Importantly, the potential effects of target detection cannot explain the memory difference between the No-go items in the motor vs. cognitive task. Some previous studies provided a possibility of memory enhancement for the No-go cues in specific situations. For instance, relatively rare No-go cues could induce greater memory than frequent Go cues (Makovski et al., 2013). Researchers explained such observation by speculating that a cue that requires switching of response plans triggers the ABE (Swallow and Jiang, 2013). Yet, this idea predicts the trade-off between Go and No-go memory and cannot explain why in this study, both Go and No-go memory increased compared with the cognitive No-go condition. This prediction was not consistent with the comparable memory for the motor Go and No-go items observed here. Rather, the engagement in the motor No-go trials itself, especially the covert process of motor engagement, seemed to improve memory encoding. Notably, it remains unclear which covert process in motor engagement was critical for the enhancement. With task-relevant memory, Kinder and Buss (2020) attributed a similar contrast between motor and cognitive task memory to the requirement of action preparation. However, besides action preparation, the motor No-go condition should be accompanied by the planning and selection of action. For instance, the inhibition of action might drive cognitive resources and boost concurrent memory compared with a motor-neutral baseline. Although we provided a concept-of-proof of AIME for task-irrelevant incidental memory, further investigation is needed to identify its underlying mechanism.

Our findings are subject to at least two limitations. First, a confounding factor in this experiment is the cognitive load required by different tasks; counting and retaining the number of Go cues could have impaired memory encoding during the cognitive task (cf., Kinder and Buss, 2020). To minimize the influence of the cognitive load, we made the number of trials in each block small (eight trials per block). Moreover, given that the difference between motor and cognitive task memories was limited to the specific (i.e., post No-go) condition, it is difficult to assume that the cognitive load impaired the memory performance throughout the cognitive task. Thus, although the influence of cognitive load should be further investigated, we believe that it was not critical for our main findings. Second, the sequential presentation of pre- and post-items might induce interference in the memory. This presentation manner was originally designed to consider the unreliable effect of the action on memory before execution. As expected, we observed memory enhancement only for the post-items and not for the pre-items. However, the presentation of post-items might mask the sensory representation of the pre-item and impede its encoding. This may have concealed the effects of motor engagement in the pre-item in the current and previous studies (Shimane et al., 2021). Future research can examine this possibility by presenting only one item, which is either before or after the action execution (i.e., keypress) in a trial.

As a final note, this study has several practical limitations. First, as our experiment was conducted in an online environment; it was less controlled compared to a laboratory experiment. It is possible that some participants were distracted during the task and there were some artifacts due to the devices or internet connection. Furthermore, the age of our participants was on average higher and more varied than in previous studies with young adults (e.g., Yebra et al., 2019; Kinder and Buss, 2020). However, our participants showed a memory performance comparable to the previous study (Yebra et al., 2019), indicating that they performed the task appropriately. Although we believe that the variability in the samples and experimental settings of this study contributes to the generalizability of our findings, the possibility that these factors may have led to differences from previous studies, such as the absence of AIME, is worth considering.

In conclusion, this study demonstrated motor-specific memory enhancement, which is not explained by the classic ABE. Nevertheless, action execution does not appear to play a critical role. Engaging in the motor task itself is sufficient to increase incidental memory for the task-irrelative items compared to a passive viewing situation. To date, most studies have examined memory in physically restricted situations. In contrast, our evidence indicated that the action automatically modulates the memory through online interaction, emphasizing the importance of further examination of motor-mnemonic interaction. This may expand on our existing understanding of everyday memory, such as active learning, for example, the advantage of active interaction between teachers and students over passive learning.

## Data availability statement

The datasets presented in this study can be found in online repositories. The names of the repository/repositories and accession number(s) can be found below: https://osf.io/9gbxq/?view_only=a3d21d84c3a24199b487d5569e271cdc.

## Ethics statement

The studies involving human participants were reviewed and approved by the Institutional Review Board of the University of Tokyo. The patients/participants provided their written informed consent to participate in this study.

## Author contributions

DS and TT: conceptualization, data curation, formal analysis, and investigation. DS, TT, KT, and KW: funding acquisition. KT and KW: supervision. All authors contributed to the article and approved the submitted version.

## Funding

This work was supported by the Japanese Society for the Promotion of Science (JSPS) KAKENHI (no. JP21K20303) to DS; Grants-in-Aid by the Nakayama Hayao Foundation for Science, Technology and Culture, Japan (no. R1-B-56), and JSPS KAKENHI (no. JP20K22269) to TT; JSPS KAKENHI (no. JP18H03505) to KT; and JSPS KAKENHI (nos. JP17H00753 and JP22H00090) and Japan Science and Technology Agency (JST) Moonshot Research and Development (no. JPMJMS2012) to KW.

## Conflict of interest

The authors declare that the research was conducted in the absence of any commercial or financial relationships that could be construed as a potential conflict of interest.

## Publisher's note

All claims expressed in this article are solely those of the authors and do not necessarily represent those of their affiliated organizations, or those of the publisher, the editors and the reviewers. Any product that may be evaluated in this article, or claim that may be made by its manufacturer, is not guaranteed or endorsed by the publisher.

## Footnotes

^1^Yebra et al. (2019) did not favor the attentional account of AIME and suggested that the Go cues were as frequent as the No-go cues and thus not highly salient. However, in previous studies, the attentional boost effect occurred when targets were as common as distractors (Swallow and Jiang, 2012; Makovski et al., 2013).

^2^Based on previous work, we expected no difference between cognitive Go and No-go conditions after the target disappeared. We thus planned to aggregate these conditions into the motor-neutral one as done by Kinder and Buss (2020). This was why the number of trials were equaled between motor Go, motor No-go, and cognitive Go/No-go conditions (24 items per condition) but not between motor and cognitive tasks. Note that, however, our sample size was sufficient even after separating cognitive Go and No-go conditions (see “Participants” and “Data analysis” section).

^3^Raymond et al. (1992) showed that the detection of the second target was suppressed for ~500 ms after that of the first target in the rapid serial visual presentation task.
