# Supplementary material for: Motor engagement enhances incidental memory for task-irrelevant items
Source: Front Psychol. 2022 Aug 16;13:914877. doi: 10.3389/fpsyg.2022.914877 (PMC9453871; doi:10.3389/fpsyg.2022.914877)
Supplement: Supplementary file 2 [file Table_2.docx]

*Table S2. the results of three-way within-participant ANOVA*

| Predictor | *df_Num_* | *df_Den_* | *SS_Num_* | *SS_Den_* | *F* | *p* | η^2^_g_ |
| --- | --- | --- | --- | --- | --- | --- | --- |
| (Intercept) | 1 | 141 | 20.83 | 11.28 | 260.41 | .000 | .46 |
| Task type | 1 | 141 | 0.04 | 2.00 | 3.04 | .084 | .00 |
| Cue type | 1 | 141 | 0.02 | 1.77 | 1.82 | .180 | .00 |
| Onset | 1 | 141 | 0.90 | 2.49 | 50.98 | .000 | .04 |
| Task type x Cue type | 1 | 141 | 0.03 | 1.58 | 2.60 | .109 | .00 |
| Task type x Onset | 1 | 141 | 0.01 | 1.39 | 0.72 | .398 | .00 |
| Cue type x Onset | 1 | 141 | 0.00 | 1.91 | 0.01 | .916 | .00 |
| Task type x Cue type x Onset | 1 | 141 | 0.05 | 1.84 | 4.05 | .046 | .00 |

*Note.* *df_Num_* indicates degrees of freedom numerator. *df_Den_* indicates degrees of freedom denominator. *SS_Num_* indicates sum of squares numerator. *SS_Den_* indicates sum of squares denominator. η^2^_g_ indicates generalized eta-squared.
